# The development of ovarian carcinosarcoma from serous tubal intraepithelial carcinoma: an immunohistochemical case report with literature review

**DOI:** 10.3389/fonc.2026.1727634

**Published:** 2026-04-15

**Authors:** Paula Szlendak, Dorota Lewkowicz, Andrea Romano, Marek Semczuk, Maciej Jóźwik, Andrzej Semczuk

**Affiliations:** 1 nd IIDepartment of Gynecological Surgery and Gynecological Oncology, Lublin Medical University, Lublin, Poland; 2 Department of Pathology, Lublin Medical University, Lublin, Poland; 3 Department of Gynecology, Maastricht University Medical Centre (MUMC), Maastricht, Netherlands; 4 Department of Gynecology and Gynecologic Oncology, Medical University of Białystok, Białystok, Poland

**Keywords:** immunohistochemistry, Ki-67, ovarian carcinosarcoma, p53, serous tubal intraepithelial carcinoma

## Abstract

Ovarian carcinosarcoma (OCS) is an uncommon and highly aggressive subtype of ovarian carcinoma characterized by the simultaneous occurrence of two malignant components: carcinomatous and sarcomatous. Located at the fimbrial region of the fallopian tube, serous tubal intraepithelial carcinoma (STIC) represents a form of precursor lesion for high-grade serous carcinoma (HGSC). In the current report, we present an intriguing case study of left OCS concomitant with ipsilateral STIC. Immunohistochemical (IHC) assessment showed a similar staining pattern of the carcinomatous OCS component and STIC. In conclusion, the development of OCS from STIC should be considered. Such a situation represents both a rare phenomenon and a challenge for preoperative diagnostic protocol. A careful application of selected IHC markers, p53 and Ki-67 in particular, may substantially help not only in the differential diagnosis but also in clinicopathological algorithms based on OCS molecular profiling into HGSC-like and non-HGSC-like variants.

## Introduction

Ovarian carcinosarcoma (OCS) is an uncommon and highly aggressive subtype of ovarian carcinoma (OC) characterized by the simultaneous occurrence of two malignant components: carcinomatous and sarcomatous (1–4). The rarity of this tumor made it challenging to ascertain its incidence (3, 5). Nowadays, OCS is thought to represent less than 2% of all ovarian malignancies (1, 5–7). The median 5-year overall survival ranges from 8 to 26 months, with disease specific survival rate of 29.8% (1, 2, 7). Compared to other OCs subtypes, OCSs have a poorer outcome across all International Federation of Gynecology and Obstetrics (FIGO) stages (2, 6, 8). From the standpoint of tumor biology, immunohistochemistry, and molecular biology, OCSs are classified most accurately as a subclass of high-grade serous carcinoma (HGSC) (9, 10).

Serous tubal intraepithelial carcinoma (STIC) represents a form of precursor lesion of HGSC, particularly frequently located at the fimbrial region within the epithelial cells of the fallopian tube (11, 12). It is a rare entity, most commonly detected in *BRCA 1/2* pathogenic variant (PV) carriers undergoing risk-reducing surgery (11). It is believed that most of HGSCs originate from the fallopian tube, and the average duration of progression from STIC to fully blown HGSC is approximately 6.5 years (11). The vast majority (>70%) of HGSCs are identified at FIGO stage III or IV of the disease (12, 13). Such an outcome can be primarily attributed to the non-specific nature of clinical symptoms at the initial phase of the disease, coupled with the lack of dependable screening techniques (13–15).

The aim of the present work was to showcase a rarely described coexistence of primary OCS with STIC arising at the same anatomical site. We also analyzed selected IHC antibody stainings in both OCS components in order to compare them with STIC. The report was written in line with the SCARE 2020 guidelines for surgical case reports (16).

## Case study

### Initial presentation and management

A 71-year-old postmenopausal woman (gravida 2, para 2) was admitted to the II^nd^ Department of Gynecological Surgery and Gynecological Oncology, Lublin Medical University, Lublin, Poland, for the presence of a left pelvic mass. Her main complaints were related to digestive tract abnormalities, primarily the sensation of intestinal twisting. Her medical history included an appendectomy 40 years earlier and a laparoscopic cholecystectomy 3 years ago. Both surgeries were performed without complications. There was no family history of female genital tract malignancies. Over the past few years, the patient developed several comorbidities, including type 2 diabetes mellitus, arterial hypertension, and hypothyroidism treated effectively by her family physician.

She had a history of regular menstrual cycles from the age of 14 years until the age of 49. She was pregnant five times, with two full-term vaginal deliveries and three spontaneous first-trimester miscarriages.

In pelvic examination, a palpable mobile tumor was detected in the left adnexal region. Transvaginal ultrasound (TVUS) examination revealed the uterine corpus to be anteflexed, measuring 45×51 mm, with a homogeneous linear 2-mm-thick endometrium. A small fundal leiomyoma of 5-mm diameter was noted. A trace amount of free fluid was present in the uterine cavity. The right ovary was of normal morphology and size: 30×10 mm, yet the right fallopian tube could not be clearly delineated. In the left adnexal area, a mobile solid-cystic ovarian mass measuring 50x20 mm was observed, with the fallopian tube length measured to be approximately 50 mm.

In preoperative work-up, serum CA125 level was elevated to 66.4 U/mL (normal value: ≤30 U/mL), whereas other serum cancer markers and standard diagnostic tests were within their normal limits.

An exploratory laparotomy was performed. Peritoneal fluid was collected for cytological examination. Intraoperatively, a tumor originating from the left adnexa, approximately 50-mm in diameter, was confirmed. The tumor was completely removed for immediate anatomopathological verification, and microscopically negative surgical margins (R0 resection) were confirmed. Upon palpation and macroscopic evaluation, the uterine corpus, right adnexa, and remaining abdominal organs appeared unremarkable. Altogether, the patient underwent total abdominal hysterectomy, bilateral salpingo-oophorectomy, appendectomy, omentectomy, peritoneal lavage, and pelvic and para-aortic lymph nodes dissection. This surgery was performed with no complications, and the patient was discharged home on the fourth postoperative day.

Later, she was admitted to the Oncology Department at the Lublin Oncology Center, Lublin, Poland, where a chemotherapy protocol consisting of carboplatin, paclitaxel, and bevacizumab was applied. After the completion of 6 cycles of chemotherapy, an abdominal contrast-enhanced computed tomography (CT) was performed and no recurrent disease was found. The patient still remains under close oncologic surveillance and is disease-free. Repeat imaging by TVUS and abdominal CT done two years following surgery showed no evidence of disease. No elevation of tumor markers was observed thereafter. Her current follow-up is 3.5 years.

### Final anatomopathological examination

**Figure 1:**
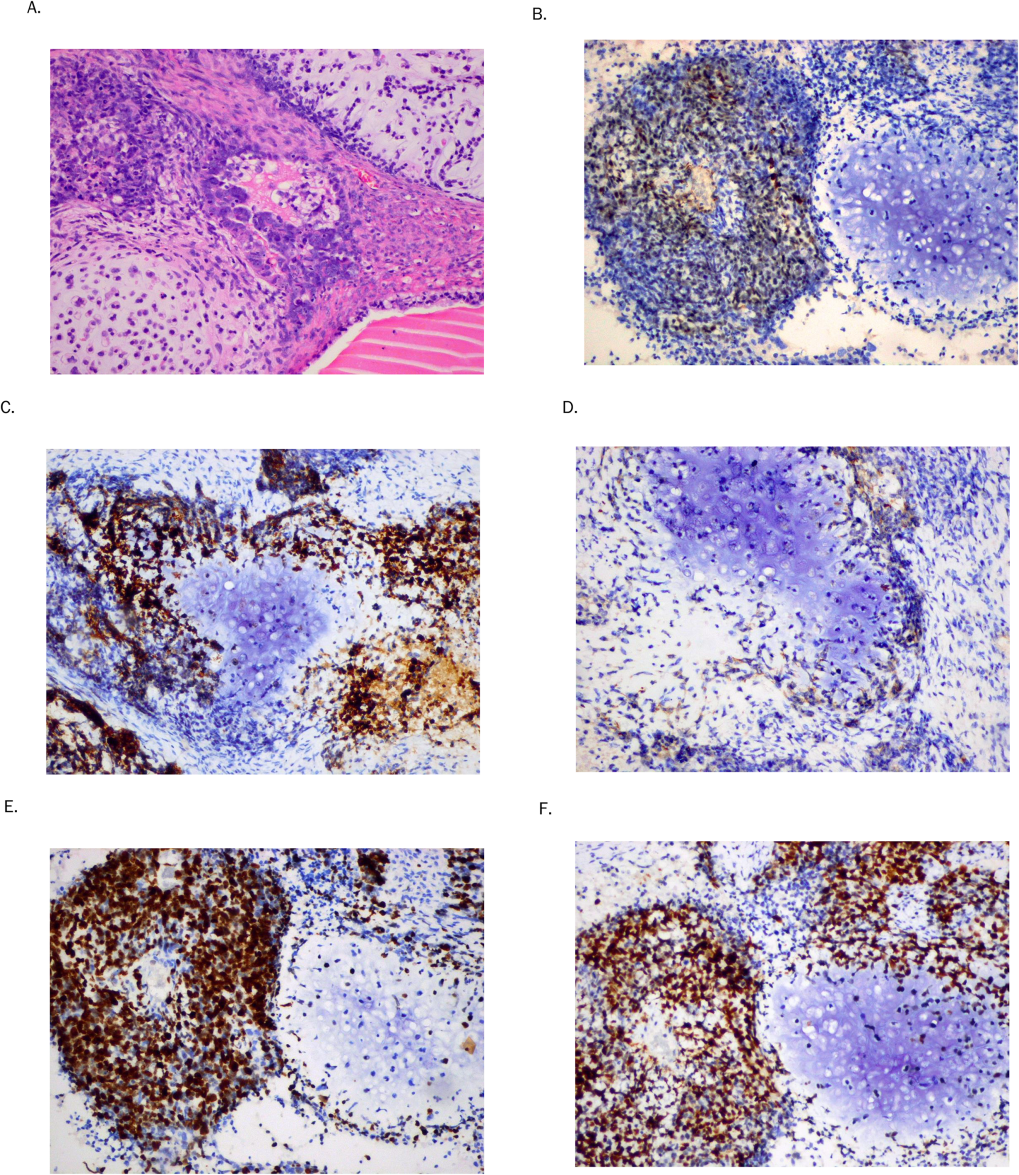
Immunohistochemical staining of hematoxylin and eosin **(A)** in OCS. Selected IHC markers: WT1 **(B)**, CK7 **(C)**, CK20 **(D)**, Ki67 **(E)**, and p53 **(F)** in ovarian carcinosarcoma (x100).

**Figure 2:**
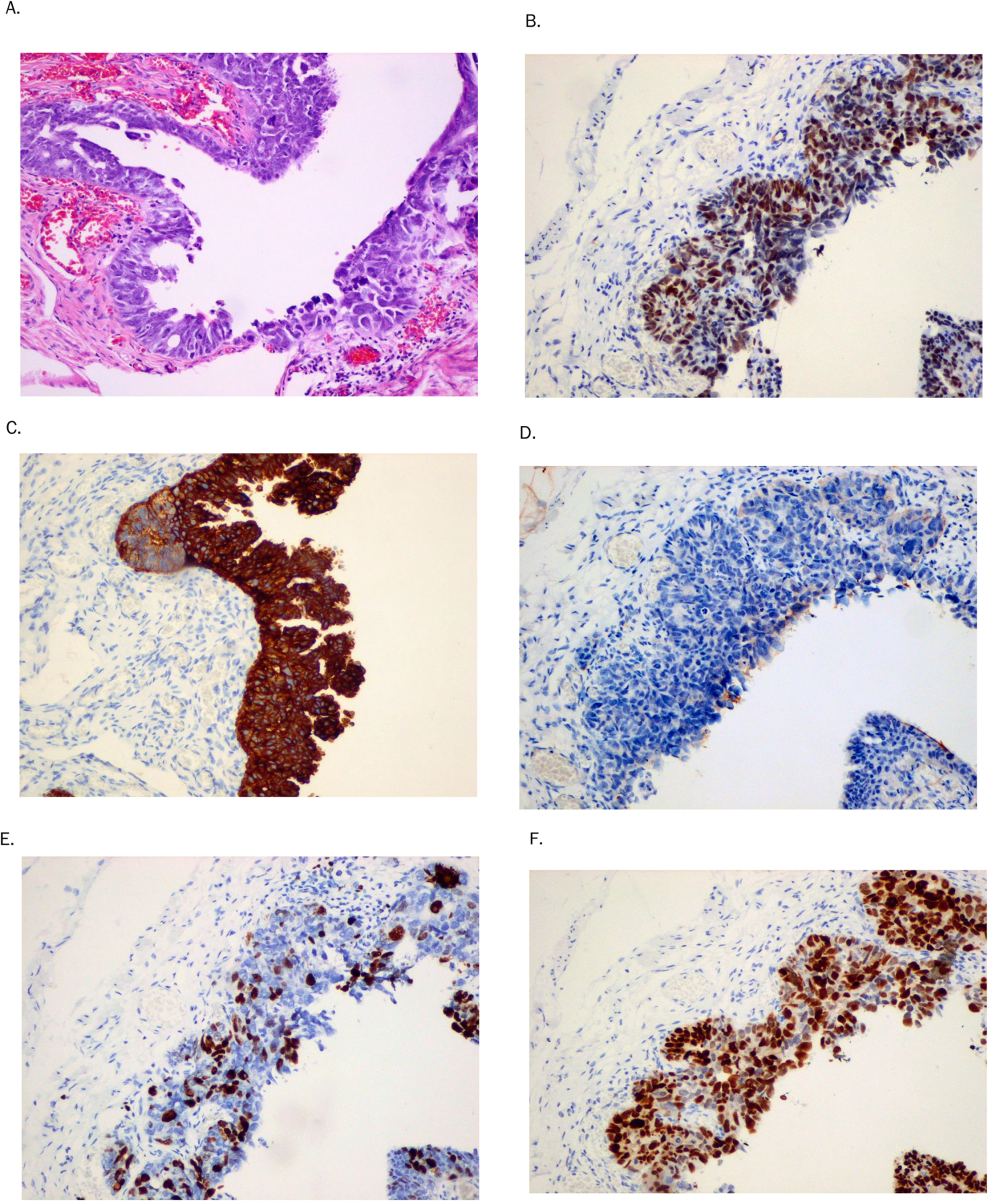
Immunohistochemical staining of hematoxylin and eosin **(A)** in STIC. Selected IHC markers: WT1 **(B)**, CK7 **(C)**, CK20 **(D)**, Ki67 **(E)**, and p53 **(F)** in serous tubal intraepithelial carcinoma (x100).

The postoperative anatomopathological examination revealed primary left OCS consisting of HGSC, clear-cell carcinoma, and heterologous sarcoma composed of G2 chondrosarcoma (**Figure 1A**). The tumor tissue was also present on the outer surface of the ovary. Neither angioinvasion, nor lymphovascular space invasion (LVSI) were found. The peritoneal fluid was free of neoplastic cells. Cross-sections of the left fallopian tube showed cancer infiltration on the outer surface. In the inner tubal epithelium, focal changes specific for STIC were detected (**Figure 2A**). Of interest, both malignancies were located in direct proximity to each other. The post-operative diagnosis was FIGO stage IA ovarian carcinoma (17).

### Immunohistochemical assessment

The staining of 10 carefully selected immunohistochemical markers in both OCS components, as well as in STIC is presented in **Table 1**. Data concerning the antibodies used, clones, sources, dilutions, and manufacturers are shown at **Table 2**. This IHC analysis showed that the carcinomatous component was positive for WT1, CK7, WT1, CK20 (4%), napsin (8%), Ki67 (90%) and p53 (90%) (**Figures 1B–D**; **Figures 2B–D**, respectively). S100 was positive in the chondrosarcoma OCS component only, and SMA was positive in 70%. Interestingly, mere 10% of sarcomatous cells were positive for Ki-67 and there was only focal (30%) p53 positivity present in the sarcomatous component, in contrast to the much more pronounced immunoreactivity for Ki-67 and p53 in both the carcinomatous OCS component and STIC (**Figures 1E,F**; **Figures 2E,F**, respectively). Finally, it is worth underlining that we previously performed several experiemental research of various IHC markers assessment in female genital tract carcinosarcomas, especially in uterine carcinosarcomas (18–20).

**Table 1:** Staining patterns of selected immunohistochemical markers in both OCS components compared to STIC. (+), positive (–);, negative. .

Immunohistochemical marker	OCS component		STIC
	Carcinomatous	Sarcomatous	
WT1	+	–	+
CK7	+	–	+
CK20	focally + (4%)	–	–
S100	–	+ (in chondrosarcoma)	–
Caldesmon	–	–	–
SMA	–	+ (70%)	–
P40	–	–	–
Napsin	focally weakly + (8%)	–	–
Ki67	+ in about 90%	+ in about 10%	+ in about 50%
p53	+ in most cells (90%) (=mutated)	+ focally (30%)	+ in most cells (90%) (=mutated)

**Table 2:** Brief description of the antibodies used in the experiments.

IHC marker	Antibody	Clone	Dilution	Manufacturer
WT1	Monoclonal mouse anti-human	6F-H2	RtU*	DAKO
CK7	Monoclonal mouse anti-human	OV-TL 12/30	RtU	DAKO Omnis
CK20	Monoclonal mouse anti-human	K_5_ 20.8	RtU	DAKO Omnis
S100	Polyclonal rabbit	Anti-S100	RtU	DAKO
Caldesmon	Monoclonal mouse anti-human	IR054	RtU	DAKO
SMA	Monoclonal mouse anti-human	h-CD	RtU	DAKO
P40	Monoclonal rabbit anti-human	1A4	RtU	Aligent
Napsin	Monoclonal mouse anti-human	MRQ-60	1:200	Cell Marque
Ki67	Monoclonal mouse anti-human	MIB-1	RtU	DAKO Omnis
p53	Monoclonal mouse anti-human	DO-7	RtU	DAKO Omnis

^Ready-to-Use.^

## Discussion

In the literature, STIC has been proposed as a precursor lesion of some OCSs, namely, of subtype II tumors of HGSC-like morphology (11, 21–24). Weinberger et al. (11) reported that “…many type II (ovarian) carcinomas developed from STIC predominantly in the distal portion of the fallopian tube and it is very likely to point of the origin of a significant subset of the pelvic high-grade serous carcinoma …”. In addition, STIC may be a precursor entity of many primary female genital tract carcinosarcomas, such as those originating predominantly from the peritoneum (25, 26) or fallopian tube (27). Of note, STIC may be present independently in both fallopian tubes (28). However, the question arises whether STIC can be a precursor of both OCS components, or only of the carcinomatous component.

To date, several reports focused on the existence of possible clonal relationship between pelvic/ovarian OCSs and STIC (29–32). Kuhn et al. (29) support a clonal relationship between pelvic HGSCs and STIC. Moreover, data by Ardighieri et al. (30) showed similar p53 immunoreactivity in all bilateral OCS cases as in carcinosarcoma-associated STICs with *TP53* alterations. Identical *TP53* mutations were detected in 2 out of three (66%) HGSCs and bilateral STICs (31). A separate report, See et al. (32), found that OCS components (such as HGSC and chondrosarcoma) and STIC displayed the same *TP53* PVs, with concomitant diffuse positive p53 and p16 immunostaining. Based on the data discussed above, as well as from our IHC results, one could conclude that both carcinosarcoma components may arise from STIC. Interestingly, the chondrosarcoma component is thought to develop from fallopian tube carcinoma *via* the epithelial-mesenchymal transition process (33, 34). A propensity of the epithelial component of carcinosarcoma toward the transition into sarcomatous differentiation was proposed by Amant et al. (33), entirely in support of the monoclonal theory of histogenesis.

Compared to others antibodies, p53 and Ki-67 immunoreactivity in OCS and STIC cases was studied extensively worldwide (23, 27, 30, 35–37). From a review, positive IHC staining for p53 and Ki-67 in STIC was reported in 21 (54%) and 19 (49%) out of 39 papers verified, respectively (23). Five studies considered aberrant p53 reactivity together with increased Ki-67 staining to be “… a prerequisite for diagnosing STIC.” However, “… the other 5 studies described IHC as being supportive, but not necessary for the diagnosis …” (23). Further, Leonhardt et al. (35) suggested that “… immunohistochemistry for p53 and Ki-67 may aid in the diagnosis, but is not necessary for routine investigation ….”.

Recently, Dhillon et al. [10] divided OCSs into *p53*-mutated (HGSC-like) and *p53* wild-type (non-HGSC-like) variants and recommended routine p53 IHC staining in all cases. Notably, although most of their OCS cases showed morphologic and molecular profiles resembling HGSC, some of them suggested their origin *via* the non-serous pathway. The p53 status has strong clinical implications with regard to patients’ survival (10). Finally, these authors advocated that “ … all HGSC-like OCSs should be sent for *BRCA1/2* testing to identify patients eligible for PARPi therapy…” (10). PARPi therapy is an established effective treatment with Poly (ADP-ribose) polymerase (or, PARP) inhibitors of *BRCA1/BRCA2* mutant carriers.

In our present study, substantial differences in selected staining patterns were reported between particular OCS components (see Results). Significantly, both the cancerous OCS component and STIC displayed similar p53 staining pattern, typical for HGSC-like group tumors. Furthermore, one of the novelties of our study was to demonstrate the immunostaining pattern in OCS and STIC of the markers that have never been investigated in these tumors before (see Results).

In the literature, several reports analyzed some other IHC markers within STIC lesions (37–41). For example, STIC was found to express Rsf-1, cyclin E, and fatty acid synthase, whereas mucin-4 staining did not change or even was reduced in the majority of the cases (38). Moreover, Kuhn et al. (39) reported that laminin γ1 expression was significantly higher (*p* < 0.001) in STIC and HGSC compared with normal fallopian tube epithelium. Novak et al. (40) showed that stathmin and p16^INK4A^, when investigated together with p53 and Ki-67, improved the differential diagnosis of STIC. They could thus be widely applied as sensitive adjunct markers in both p53-positive and p53-negative cases (40). An array of novel proteins (UCHL1, ADAMTS13, and GAPDH) and immune response markers (CD4+ cells and FOXP3+ T regulatory cells) proved altered in STIC lesions (41). Finally, Japanese researchers (37) found that “CD44v9 loss contributes to the stepwise progression of p53 signature to dormant STIC …”. Let us suggest that this new IHC staining should be added to the diagnostic protocols when large observational studies confirm this interesting finding.

The diagnosis of OCS is possible after careful assessment of post-operative pathological material because these tumors constitute only up to 1.8% of all ovarian malignancies (1, 5, 42). Moreover, preoperative symptoms are scarce and non-specific, and the disease is mostly diagnosed as an advanced high-grade ovarian neoplasia (1, 10, 24). The management of OCS is similar to that of HGSCs, consisting of cytoreductive surgery combined with adjuvant systemic treatment by chemotherapy and PARPi (34, 42). Unfortunately, OCS patients’ outcome is rather fatal since most of them die within 2 years from the initial diagnosis (1, 5). This outcome is even worse compared with those observed for primary epithelial ovarian cancer or uterine carcinosarcoma (5, 8). Besides, data regarding the management of solitary STICs are very limited and varied for different countries (43, 44).

The final question arises whether all salpingectomy specimens should undergo routine IHC assessment for the detection of early STIC lesions. In 2022, Vang and Shih (13) reported that even “half of all carcinomas identified in risk-reducing salpino-oophorectomy specimens were in the form of STIC…”. However, STIC may also be incidentally detected in patients with a low or average risk for ovarian/tubal/peritoneal carcinomas (13). In recent publications, precise histologic examination of the fallopian tubes, with a particular emphasis on the fimbiae, in all salpingectomy specimens has been recommended (45, 46). Such an approach offers the advantage to detect early (microscopic) malignant changes in the tubal epithelium (45). And we humbly concur with this suggestion.

## Conclusions

Collectively, the development of OCS from STIC is a possible yet rather rare phenomenon. The initial (preoperative) diagnosis can be a significant challenge for clinicians. A scrupulous application of selected IHC markers, p53 and Ki-67 in particular, has a potential to substantially help not only in the differential diagnosis but also in OCS molecular profiling into HGSC-like and non-HGSC-like groups.

## Data Availability

The raw data supporting the conclusions of this article will be made available by the authors, without undue reservation.
